# Analysis of *Gln223Agr* Polymorphism of Leptin Receptor Gene in Type II Diabetic Mellitus Subjects among Malaysians

**DOI:** 10.3390/ijms140919230

**Published:** 2013-09-18

**Authors:** Ali Etemad, Vasudevan Ramachandran, Seyyed Reza Pishva, Farzad Heidari, Ahmad Fazli Abdul Aziz, Ahmad Khairuddin Mohamed Yusof, Chong Pei Pei, Patimah Ismail

**Affiliations:** 1 Genetic Research Group, Department of Biomedical Science, Faculty of Medicine and Health Sciences, Universiti Putra Malaysia, Serdang, Selangor 43400, Malaysia; E-Mails: ali_etemad_c@yahoo.com (A.E.); reza_xe54@yahoo.com (S.R.P.); farzad720@gmail.com (F.H.); 2 Institute of Gerontology, Universiti Putra Malaysia, Serdang, Selangor 43400, Malaysia; E-Mail: vasuphd@gmail.com; 3 Department of Medicine, Faculty of Medicine and Health Sciences, Universiti Putra Malaysia, Serdang, Selangor 43400, Malaysia; E-Mail: afazli@upm.edu.my; 4 Department of Cardiology, National Heart Institute, Kuala Lumpur, Selangor 50400, Malaysia; E-Mail: ahmadk@ijn.com.my; 5 Department of Biomedical Science, Faculty of Medicine and Health Sciences, Universiti Putra Malaysia, Serdang, Selangor 43400, Malaysia; E-Mail: cpp@upm.edu.my

**Keywords:** leptin receptor, polymorphism, type 2 diabetes mellitus, Malaysia, restriction enzyme

## Abstract

Leptin is known as the adipose peptide hormone. It plays an important role in the regulation of body fat and inhibits food intake by its action. Moreover, it is believed that leptin level deductions might be the cause of obesity and may play an important role in the development of Type 2 Diabetes Mellitus (T2DM), as well as in cardiovascular diseases (CVD). The Leptin Receptor (LEPR) gene and its polymorphisms have not been extensively studied in relation to the T2DM and its complications in various populations. In this study, we have determined the association of *Gln223Agr* loci of LEPR gene in three ethnic groups of Malaysia, namely: Malays, Chinese and Indians. A total of 284 T2DM subjects and 281 healthy individuals were recruited based on International Diabetes Federation (IDF) criteria. Genomic DNA was extracted from the buccal specimens of the subjects. The commercial polymerase chain reaction (PCR) method was carried out by proper restriction enzyme *MSP I* to both amplify and digest the *Gln223Agr* polymorphism. The *p*-value among the three studied races was 0.057, 0.011 and 0.095, respectively. The values such as age, WHR, FPG, H_b_A_1C_, LDL, HDL, Chol and Family History were significantly different among the subjects with *Gln223Agr* polymorphism of LEPR (*p* < 0.05).

## 1. Introduction

Hasty socioeconomic development has resulted in significant changes in the lifestyle of many, which has made diabetes a growing concern in many developing countries. According to the Malaysian Third National Health Morbidity Survey in 2006 [1], the prevalence of Type 2 diabetes mellitus (T2DM) among adults was found to be 14.9%. Obesity and insulin resistance are believed to be linked to the metabolic syndrome (MS), a group of risk factors for cardiovascular diseases (CVD) and T2DM.

It has been proven that increased Body Mass Index (BMI) accounts as a strong risk factor for T2DM [2–5]. Besides, a positive association between obesity and T2DM has been documented both in women and men [2,4] with increased risk of developing insulin resistance.

There are many genes that interact with the environment that lead to obesity or diabetes and these genes have been investigated to be involved in determining the whole range of BMI in the population [6].

It is believed that CVD could be the leading cause of death among T2DM patients, which accounts for up to 70% of their mortality [7]. Furthermore, patients with T2DM share the same risk of myocardial infarction compared to individuals who have already suffered from a heart attack and this leads many clinicians to consider that the T2DM is equivalent to CVD [8]. The death rate among the CVD patients with T2DM was also falling [9]. On the other hand, the prevalence of T2DM has increased rapidly and the concern is that the prevalence of CVD might also start to increase in agreement with that. Therefore, the beginning of aggressive CVD in patients with T2DM is essential as it might be triggered by the elevated lipid levels (in particular Triglyceride & cholesterol) [10].

Leptin, which is also known as adipose tissue peptide hormone, plays an important role in the regulation of body fat and, therefore, it is called the obesity hormone [11]. Since leptin inhibits food intake through its action, it is believed that reduced leptin levels might be the cause of obesity [12] and indeed, several obese individuals were identified with low leptin levels [13]. However, several studies have reported that in most obese individuals, leptin levels were either normal or higher than normal individuals [14].

These results indicate that among obese individuals, there might be some kind of leptin resistance which is believed to play an important role in the development of obesity and this is due to the fact that an increased amount of leptin cannot perform its role in controlling food intake [15,16].

Leptin resistance is believed to occur as a result of genetic defect in the leptin receptor gene (LEPR), where the element of the gene lacks most of the cytoplasmic region; hence, individuals with this mutation tend to eat more than needed and keep gaining weight [17]. Abnormal LEPR, as well as abnormal leptin catabolism, has been detected in obesity development [18].

The monogenic form of obesity comes from the mutations in a certain loci which then cause obesity. Obesity is also known as the multifactorial disorder which depends on multiple genes and their contribution (polygenic). An individual may carry the mutated gene that may not have developed obesity in suitable environment [19].

In relation to obesity and T2DM, LEPR gene polymorphisms were not extensively studied among different populations. However, to our knowledge, the association of the Gln223Agr loci of LEPR gene has not been investigated among Malaysian diabetic subjects. Taking this into account, this study was initiated to determine the association of the selected polymorphism of the LEPR gene between T2DM subjects and healthy individuals among the three ethnic groups in Malaysia; Malays, Chinese and Indians.

## 2. Results and Discussion

In this study, the Gln223Agr loci was investigated between T2DM patients and healthy controlled group among the three main ethnic groups in Malaysia; Malays, Chinese and Indians and a significant difference had been found in the genotype frequencies between the Chinese T2DM and the Chinese controlled subjects.

Approximately 600 subjects were approached and 565 subjects were included in this study, whereby the outliers and missing values were excluded due to skewness value. This total included 284 (50.3%) T2DM subjects who are comprised of 191 (67.3%) males and 93 (32.7%) females, followed by 281 healthy subjects (49.7%) with 158 (56.2%) males and 123 (43.8%) females. Overall, the majority of the subjects were males, 349 (61.8%). The age of the subjects ranged from 36 to 85 years old, with a mean of (61.9 ± 9.8) for T2DM cases and as for the controlled group, their age ranged from 30 to 84 years old, with a mean of (53.3 ± 12.4). Nearly 43.9% of the subjects have family history of T2DM or CVD; a total of 139 reports (49.5%) among the T2DM subjects and 107 reports (38.2%) among the controlled group.

The basic characteristics of the two groups subjected to this study are shown in Table 1. The significant differences were found in Age, Body Mass Index (BMI), Waist Hip Ratio (WHR), Systolic Blood Pressure (SBP), Fasting Plasma Glucose (FPG), H_b_A_1C_, High Density Lipoprotein (HDL), Cholesterol (Chol), Triglyceride (TG) and CVD Risk% between the selected groups (*p* < 0.05). Other values differed significantly in T2DM subjects when compared to the controlled group such as Family history. However, the Diastolic Blood Pressure (DBP) and Low Density Lipoprotein (LDL) levels did not differ significantly (*p* > 0.05) as described in Table 1. The age, BMI, WHR, SBP, DBP, FPG, H_b_A_1C_ and TG of the variables at the time of participation were significantly higher in the DM group than the controlled category (*p* < 0.05), however, the levels of LDL, HDL and Chol were higher in the controlled group.

High levels of cholesterol cause several health problems such as CVD, atherosclerosis and gall stones. The main step, which triggers the development of atherosclerosis, is believed to be the deposition of cholesterol-ester which fills the macrophage foam cells and contribute to the formation of the atherosclerotic plaque [20]. Gall stones are formed in the bile of obese people and they are saturated with cholesterol [21]. The cause of this saturation is the increased hepatic production of cholesterol, as well as low HDL levels [22]. Elevated plasma levels of TG are discovered in both obese and T2DM non-insulin-dependent subjects who are associated with CVD development [23].

The link between obesity, diabetes and TG is believed to be the pancreatic β-cell dysfunction which is caused by excessive amounts of TG [24]. A study has revealed the role of leptin and the reduction of TG levels in all cells, which expresses the leptin receptor on its surface “via inducing an increase in Free Fatty Acid (FFA) oxidation and a decrease in esterification” and this action could be absent in obese subjects due to leptin resistance [24]. The overall TG level between the T2DM subjects and the healthy individuals was significant (*p* = 0.001) but as far as the Gln223Agr genotypes are concerned, it was no more significant in our observation (*p* = 0.139). Biochemical factors have been evaluated regardless of the type of drugs, dosage and duration of medicine consumption. The present study has to be interpreted within the context of its limitations. This confounding pattern might be due to the medications taken by the patients in lowering their blood pressure and lipid levels and this could be considered as a limitation in the study. Also, the present study provided only the evidence of the association between the genetic polymorphism of LEPR gene and T2DM at the gene level. However, replication studies are much needed to confirm the association of genetic polymorphisms of LEPR gene among T2DM and their complications.

### 2.1. Genotypic and Allelic Frequency

**Figure 1:**
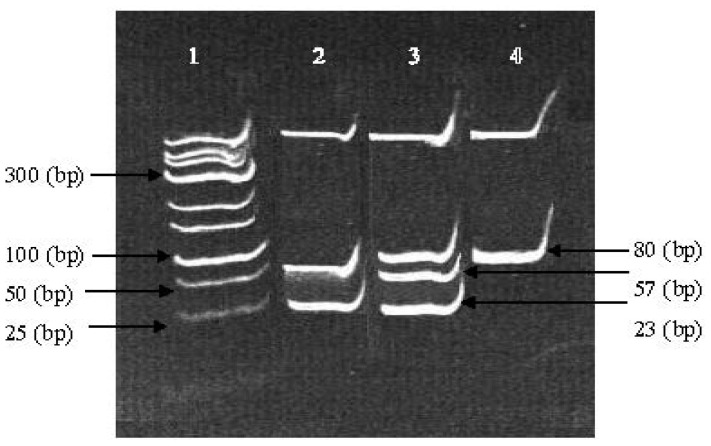
The Enzymatic Digestion of Gln223Agr loci with (*MSP I*) on 8% PAGE. Lane 1 is the DNA marker, lane 2 is the homozygous mutant and lane 3 is the heterozygous whereas lane 4 is the wild type.

In this study, three bands were detected for Gln223Agr loci, 80 (bp) which represent the Wild Type (WT) and (GG) genotype, the 57 and 23 (bp) presence the Homozygous (*HOM*) genotype (AA) followed by the presence of all three bands 80, 57 and 23 (bp) for Heterozygous (*HET*) pattern (AG), as shown in Figure 1. The genotypic and allelic frequencies of the Gln223Agr loci were deviated from Hardy-Weinberg equilibrium for different ethnic groups due to the differences between the observed and the expected results.

The comparison between the T2DM patients and the healthy individuals in this study looked into the three races, which is split into three sub-groups (Table 2). The genotype and allele frequency of LEPR-Gln223Agr was significant for T2DM/controlled Chinese group only (*p* = 0.011) but, it was not significant (*p* = 0.086) between the Chinese males and females. The homogeneity test using *Leven statistic* was significant (*p* = 0.000) and this emphasized that the error in variance was not equal in both the groups.

The overall allele frequency for Gln223Agr polymorphism among the T2DM subjects and the controlled Malays and Indians were not significant; *p* = 0.057 and 0.095, respectively. Besides, the biochemical analyses with the impact of Gln223Agr polymorphism was performed based on 2 way ANOVA and is shown in Table 3. The results show that there was a significant difference between the T2DM patients and the healthy individuals in terms of age, WHR, FPG, H_b_A_1C_, LDL, HDL, Chol and family history (*p* < 0.01). Due to the significant differences between the case and the controlled groups, a post hoc test was carried out to evaluate the probable differences among the genotypes in these two groups. The post hoc test for the Chinese race was carried out and was significantly different between wild type and homozygote (*p* = 0.019), followed by wild type and heterozygous (*p* = 0.007) and it was not significant between homozygous and heterozygous (*p* = 0.091).

In addition, the post hoc test between the overall case and the control population (without the impact of the three races) was conducted and it was significant between the wild type and Homozygote (*p* = 0.001), followed by wild type and heterozygous (*p* = 0.001) and it was not significant between homozygous and heterozygous (*p* = 0.209), as described in Table 2.

**Figure 2:**
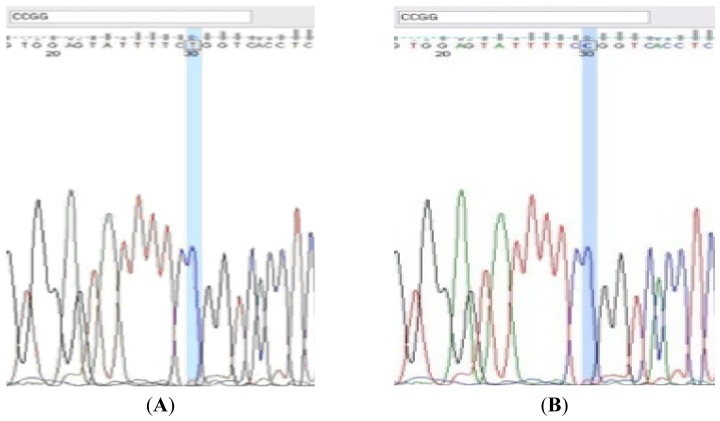
The sequencing results of the Gln223Agr loci were highlighted with the cutting site of the enzyme. The homozygous pattern (GG) was observed in (**A**) followed by wild type pattern (AA) in (**B**).

Furthermore, in order to confirm the genotype pattern, the selected samples which represented the different banding prototypes were purified after PCR amplification (Favorgene™ PCR Clean-up Kit, Wembley, Australia) and were sent for sequencing (Figure 2).

The Gln223Agr polymorphism was associated with different blood biochemical factors and anthropometric values such as age which has the youngest category in T2DM/controlled HET genotype; (52.3 ± 14.9) and (60.2 ± 9.9) respectively and FPG level, with the lowest value in both T2DM and controlled groups by HET pattern; (7.58 ± 3.1) and (5.03 ± 0.91) respectively. The H_b_A_1C_ in T2DM and WT has the lowest value; (8.13 ± 1.7), compared to the highest in WT genotype in healthy individuals; (5.83 ± 0.46). The results are proven to be the same with LDL, which was the lowest in HOM genotype of diabetes; (2.32 ± 0.74) and the highest in HOM genotype in controlled group; (2.82 ± 0.97), as portrayed in Table 3. The TG levels were not significant between the groups but, as for the HET genotype, the both groups showed the lowest value; (1.36 ± 0.64) and (1.14 ± 0.54), respectively. Despite the positive effect of HDL cholesterol and its role in lowering the risk of CVD, the overall value of HDL was lower in the diabetes group when compared to the healthy individuals. This might be due to the medications, such as *Statins*, that is prescribed routinely for the T2DM/CVD patients to not only lower their LDL and Cholesterol levels, but at the same time, to reduce other lipid components such as the HDL level and this is considered as a limitation in this study and would be worthwhile to investigate in the future on the impact of different drugs and their dosages and the period of medicine consumption.

Thus, leptin could be the final product of the obesity gene which is mainly produced by adipose tissue and involved in food intake and energy expenditure [13]. Leptin mediates its action through its interaction within a receptor which is mainly found in the hypothalamus [18]. Leptin levels are also known as the signal of body fat stores which could be affected by gender and are represented in higher amounts in females due to the different fat distribution. The overall BMI level in the T2DM females was higher in this study; (28.29 ± 5.66) compared to the males; (27.47 ± 4.38) and the BMI level was higher in the diabetic subjects compared to the controlled group (Table 1). The significant results in the genotype frequency in the study were similar to those found in other studies which associated body composition and obesity [25], insulin level and Glucose metabolism [26] and BMI [27]. However, there was no association between obesity [28], Waist circumference and BMI [29] among the Romanian obese subjects [30]. Meta-analysis studies [31,32] were failed to find out the positive and significant associations between obesity and the studied LEPR polymorphisms.

Based on the Hap map project and the advance technologies/studies, it has been recommended to select genes based on clustered regions. Due to the demographic models that include a variety of bottleneck patterns for East Asians or Europeans, the models of constant size with recent growth would be more interesting to investigate [33,34]. Since the previous genetic studies have indicated that the demographic models show as much variance as the real data [35] followed by the recent obesity growth in Asian developing countries, the genes and molecules, which regulate obesity mechanisms would be more effective to focus on.

## 3. Methodology

Ethical approval has been obtained from the ethical committee of the Faculty of Medicine and Health Sciences, Universiti Putra Malaysia (UPM) [Ref. No. JSB_Mac (12)02] and followed by the National Heart Institute (IJN) Kuala Lumpur [Ref. No. IJNEC/05/10 (02)].

### 3.1. Study Population

A case-control study was conducted to assess the patients’ medical records at hospitals and several public surveys. The cases were patients with T2DM seeking medical assistance at IJN from December 2010 to June 2012. During the study period, more than 600 subjects were approached and 565 were selected who include 284 T2DM subjects. All the case subjects (≥30 years) were selected based on International Diabetes Federation (IDF) criteria which confirmed T2DM on their medical records. Those with diabetes type 1, cancer, or any kind of serious phenotypic genetic malformation or those who were suspicious to pregnancy were excluded. A total of 281 unrelated healthy individuals were randomly recruited from different surveys in public areas and were matched with the cases by age, gender and race. The controlled subjects were free of T2DM and CVD symptoms/history at the time of participation. A questionnaire in both Malay and English languages were distributed to assess the socio-demographic factors, family history of T2DM and co-morbidities. Written informed consent was obtained from all the subjects who were participating in this study. Besides, anthropomorphic measurements too were obtained from all the subjects. Blood pressure was measured on the right arm of the subjects using an automated blood pressure monitor (Omron, Kyoto, Japan) after they were seated and rested for 5 min.

### 3.2. Sampling Method

The disposable cytobrush oral swab was used to collect the buccal specimens from the patients and kept in Phosphate Buffer Saline (PBS) at room temperature (25 °C) for further DNA extraction. The appropriate biochemical results were extracted through the profile records of the patients and were used for further evaluations and furthermore, proper biochemical tests were carried out for the controlled group since no records were obtained. The weight and height of each individual was recorded to calculate the BMI using the formula of Weight (kg)/[Height (m) × Height (m)] Next, plasma samples were analyzed on a Hitachi-912 Autoanalyser (Hitachi, Erkrath, Germany) using the respective kits supplied by Roche Diagnostics (Mannheim, Germany) to determine the levels of triglycerides (TG), High Density Lipoprotein (HDL), Total Cholesterol (T-Chol) and Low Density Lipoprotein (LDL). The lipid profiles were classified under the category of The Third Report of the National Cholesterol Education Program guidelines [36]. Lastly, blood biochemical analyses were performed after collection of an overnight fasting blood (10 h) specimen.

### 3.3. Genotyping

The available commercial DNA extraction kit (Qiagen DNA extraction kit for buccal specimens) was used to extract the genomic DNA. The quality of the extracted DNA was evaluated with electrophoresis on 0.8% agarose gel and the quantity was evaluated with nanodrop device of A260 nmA/280 nm. The targeted products of the respected genes were optimized under different cycling conditions (Table 4). The PCR master mix was prepared in 1.5 mL micro centrifuge tubes. Each reaction mixture contained 15.8 μL Distilled H_2_O, 8 μL of 2× PCR buffer (G-2000, Genet Bio, Anseo-dong Cheonan-si Chungcheongnam-do, Korea), that consist of prime Taq™ DNA polymerase 1 Unit/10 μL, 20 mM Tris-HCl, 80 mM KCl, 4 mM MgCl_2_, enzyme stabilizer, sediment, loading dye, with ph 9.0 and 0.5 mM of each dATP, dCTP, dGTP, dTTP, followed by 0.1 μL (10 pmol/μL) of each upstream and downstream primer (Forward and Reverse). From the freshly prepared master mix, 24 μL aliquots were added into 0.2 mL PCR tubes. Then, 1 μL of the DNA template (100 ng/μL) was added to each tube and this makes the final volume of 25 μL. Consumables and cycling conditions were optimized properly until the respective products were amplified without any unspecific bands and they were confirmed with the published articles.

### 3.4. Enzymatic Digestion

The amplified PCR products were mixed with 2 μL of 1× Ultra Power™ loading dye (Norgen Biotek Corporation, Thorold, ON, Canada) and were separated using the standard method through the 2% agarose gel electrophoresis. Agarose gels were visualized directly under the UV light and the images were captured at Alpha Imager (Alpha Innotech, San Leandro, CA, USA) to confirm the accuracy of the amplification and the previous procedures. After that, all the PCR products were digested with the respective restriction enzyme (*MSP I*) with proper incubation procedure as mentioned in Table 4 and loaded on 8% polyacrylamide agarose gel electrophoresis (PAGE). The staining was simultaneously done by mixing 1× Ultra Power™ loading dye with digested PCR products (Figure 1). In addition, the UVIDoc Version 98 was utilized for scoring the gel electrophoresis banding pattern which was sorted in excel format prior to statistical analysis.

### 3.5. DNA Sequencing

The sequencing method was utilized in this study after detecting the polymorphisms through the PAGE. The selected polymorph samples were purified with *Favorgene* Purification Kit and sent for sequencing (Medigene Sdn Bhd, Puchong, Malaysia) to confirm the accuracy of the amplifications and scoring patterns. The sequencing results were subjected to BLAST (http://blast.ncbi.nlm.nih.gov/Blast.cgi) and it had been verified with the published loci sequences for the respective polymorphism. The two softwares MEGA 5.2 (Molecular Evolutionary Genetics Analysis version 5; http://www.megasoftware.net) and FinchTV 1.4.0 (Geospiza, Inc.; Seattle, WA, USA; http://www.geospiza.com) were utilized for evaluation purposes (Figure 2).

### 3.6. Data Validation

As described by Zeggini *et al*., 2008 [38], nearly 10% of the samples who responded to the heterozygous genotypes were chosen and the samples were genotyped for the same assay for the second time. There was 100% accuracy with the previous sample amplification results which was done with the same assay. Moreover, duplicated samples were amplified and the results were scored with other operator.

### 3.7. Statistical Methods

Statistical analyses were performed using the SPSS (Version 17.0, SPSS Inc., Chicago, IL, USA). The variables were continuously examined for skewness and the normal values were evaluated by proper parametric tests. The normality test was conducted to monitor the variability distribution and those variables which were missed or the outliers were excluded from further evaluations. A chi-square test was used to compare the frequencies of the qualitative variables in both groups and to determine if the genotype frequencies were in Hardy Weinberg equilibrium (HWE). Furthermore, descriptive statistics were utilized to compare the allele and genotype frequencies between the both groups and their significance level was at (*p* < 0.05) by Fisher exact test. The general linear model (GLM), particularly univariate ANOVA (analysis of variance), was considered to determine the impact of genotypes with each biochemical variable or anthropomorphic value. The *p*-value was used to determine the association between the significance levels (*p* < 0.05) and due to the significant genotypes; post hoc tests were conducted too. Prior to advanced analyses, the raw data was evaluated by scatter plots and due to the inconsistent distribution; the regression analysis was omitted for this study.

## 4. Conclusions

The present study implies that Gln223Agr polymorphism of LEPR gene might be considered as an independent risk factor for the development of T2DM among Malaysians particularly in Chinese ethnics. The findings of this study will serve as a basic research platform for further research to know the mechanism or the functionality of the variant of the LEPR gene. Future studies are needed to determine the significance of LEPR gene polymorphism and their true effect on drug metabolism followed by the investigation of probable side effects.

## Figures and Tables

**Table 1:** Demographic characteristics of the study subjects.

Characteristics		T2DM		Lower/Upper		Control		Lower/Upper	*p*-value

	Male	Female	Total	CI 95%	Male	Female	Total	CI 95%	
Age (years)	60.7 ± 10.2	61.4 ± 8.8	61 ± 9.8	59.9–62.2	56.1 ± 11.2	49.7 ± 13	53.3 ± 12.4	51.8–54.8	0.000 **
BMI (Kg/m^2^)	27.47 ± 4.38	28.29 ± 5.66	27.73 ± 4.83	27.1–28.3	26.42 ± 4.32	26.07 ± 4.94	26.27 ± 4.59	26.8–26.1	0.014 *
WHR	0.95 ± 0.05	0.93 ± 0.07	0.94 ± 0.06	0.94–0.95	0.92 ± 0.05	0.86 ± 0.07	0.9 ± 0.06	0.89–0.91	0.000 **
SBP (mm Hg)	140.59 ± 22.55	144.37 ± 22.63	141.83 ± 22.6	139.1–144.56	138.34 ± 20.69	134.21 ± 18.75	136.64 ± 19.98	134.24–139.05	0.048 *
DBP (mm Hg)	80.05 ± 9.89	78.25 ± 9.79	79.45 ± 9.88	78.26–80.65	79.47 ± 9.47	78.03 ± 9.4	78.87 ± 9.45	77.73–80.02	0.170
FPG (mmol/L)	8.29 ± 3.17	8.67 ± 3.4	8.41 ± 3.24	8.02–8.81	5.49 ± 0.98	4.79 ± 0.81	5.19 ± 0.97	5.07–5.31	0.000 **
H_b_A1_C_	8.22 ± 1.94	8.27 ± 1.64	8.24 ± 1.84	8.02–8.45	5.85 ± 0.51	5.7 ± 0.38	5.79 ± 0.46	5.73–5.86	0.000 **
LDL (mmol/L)	2.31 ± 0.78	2.46 ± 0.89	2.36 ± 0.82	2.26–2.46	2.74 ± 0.97	2.71 ± 0.97	2.73 ± 0.97	2.61–2.84	0.060
HDL (mmol/L)	1.11 ± 0.28	1.31 ± 0.36	1.17 ± 0.32	1.13–121	1.16 ± 0.33	1.34 ± 0.42	1.23 ± 0.38	1.19–1.28	0.000 **
TG (mmol/L)	1.61 ± 0.81	1.62 ± 0.75	1.61 ± 0.79	1.52–1.71	1.5 ± 0.83	1.04 ± 0.49	1.3 ± 0.74	1.28–1.38	0.001 **
Chol (mmol/L)	4.17 ± 0.96	4.59 ± 1.04	4.3 ± 1.01	4.18–4.43	4.56 ± 1.03	4.67 ± 1.25	4.61 ± 1.13	4.47–4.74	0.003 **
CVD Risk%	3.97 ± 1.25	3.78 ± 1.31	3.9 ± 1.27	3.75–4.06	4.15 ± 1.28	3.67 ± 1.17	3.94 ± 1.26	3.79–4.09	0.014 **
Family History									
No	97 (51.1)	45 (49.5)	142 (50.5)		104 (66.2)	69 (56.1)	173 (61.8)		0.007 **,†
Yes	93 (48.9)	46 (50.5)	139 (49.5)		53 (38.8)	54 (43.9)	107 (38.2)		
Duration (years)	10 ± 8	10.6 ± 8.2	10.2 ± 8.1		-	-	-		-

* *p*-value was calculated by χ^2^ test with 2 × 2 contingency table and considered *p* < 0.05 as significant;

** *p*-value was calculated as *p* < 0.01 level;

† Fisher Exact test Confidence Interval: CI.

**Table 2:** The genotypic and allelic distribution among the study subjects.

	T2DM (*n*-284)			Controls (*n*-281)			
	
Genotypes and Alleles	Malay (*n* = 145)	Chinese (*n* = 49)	Indians (*n* = 90)	Malay (*n* = 133)	Chinese (*n* = 71)	Indians (*n* = 77)	*p* value
WT (GG)	28.7	26.1	41.2	16.4	8.8	30.1	0.057
HOM (AA)	59.6	73.9	50.6	68.8	83.8	50.7	0.011 *
HET (GA)	11.8	-	8.2	14.8	7.4	19.2	0.095

Alleles							
G	0.346	0.261	0.453	0.238	0.125	0.397	NS
A	0.654	0.739	0.547	0.762	0.875	0.603	

Post-Hoc Test	*p* value		(95% Confidence interval(lower bound-upper bound)				

Chinese alone							
GG *vs*. AA	0.019 *		0.50–0.54				
GG *vs*. GA	0.007 *		0.19–1.14				
AA *vs*. GA	0.091		−0.05–0.81				

Three Ethnics							
GG *vs*. AA	0.001 *		0.80–0.27				
GG *vs*. GA	0.001 *		0.11–0.41				
AA *vs*. GA	0.001 *		−0.41–−0.11				

* *p* value < 0.05,

NS- Non- Significant (*p* > 0.05) between the three ethnics, WT: wild type, HOM: homozygous, HET: heterozygous.

**Table 3:** The clinical characteristics of the T2DM and control subjects with the impact of Gln223Agr loci.

Factors	T2DM			Control			*p*-value

	WT (GG)	HOM (AA)	HET (GA)	WT (GG)	HOM (AA)	HET (GA)	
Age (years)	61.8 ± 9.8	60.5 ± 9.5	60.2 ± 9.9	56.4 ± 11.2	53.8 ± 12.0	52.3 ± 14.9	0.007 **
BMI (Kg/cm^2^)	27.87 ± 5.26	27.67 ± 4.73	27.4 ± 4.31	26.43 ± 4.67	26.31 ± 4.67	26.35 ± 4.13	0.423
WHR	0.94 ± 0.06	0.94 ± 0.06	0.93 ± 0.03	0.91 ± 0.05	0.89 ± 0.06	0.9 ± 0.1	0.022 **
SBP (mm Hg)	145.6 ± 19.9	139.2 ± 23.1	144.3 ± 22.7	135.36 ± 18.65	137.8 ± 20.4	134.5 ± 19.6	0.334
DBP (mm Hg)	80.27 ± 8.7	78.97 ± 10.1	79.34 ± 7.8	77.48 ± 8.17	79.32 ± 9.82	78.52 ± 9.16	0.385
FPG (mmol/L)	8.28 ± 3.2	8.55 ± 3.27	7.58 ± 3.1	5.59 ± 0.75	5.17 ± 0.99	5.03 ± 0.91	0.000 **
H_b_A1_C_	8.13 ± 1.7	8.26 ± 1.8	8.43 ± 2.2	5.83 ± 0.46	5.79 ± 0.47	5.76 ± 0.54	0.000 **
LDL (mmol/L)	2.41 ± 0.88	2.32 ± 0.74	2.55 ± 1.22	2.57 ± 0.9	2.82 ± 0.97	2.59 ± 1.09	0.000 **
HDL (mmol/L)	1.16 ± 0.25	1.17 ± 0.35	1.12 ± 0.29	1.25 ± 0.33	1.24 ± 0.38	1.22 ± 0.42	0.003 **
TG (mmol/L)	1.54 ± 0.61	1.65 ± 0.88	1.36 ± 0.64	1.43 ± 0.71	1.31 ± 0.78	1.14 ± 0.54	0.139
Chol (mmol/L)	4.44 ± 1.13	4.25 ± 0.92	4.18 ± 1.15	4.47 ± 0.97	4.7 ± 1.15	4.43 ± 1.28	0.010 **
CVD Risk%	3.99 ± 1.23	3.9 ± 1.29	3.67 ± 0.89	3.79 ± 1.06	3.98 ± 1.28	3.82 ± 1.2	0.216
Family History							
No	37 (43.5)	86 (54.8)	11 (50)	31 (63.3)	108 (59.3)	27 (71.1)	0.000 **,†
Yes	48 (56.5)	71 (45.2)	11 (50)	18 (36.7)	74 (40.7)	11 (28.9)	
Duration (years)	10.9 ± 8.4	9.9 ± 7.9	8.6 ± 6.3				-

WT: wild type, HOM: homozygous, HET: heterozygous;

** *p*-value was calculated by χ^2^ test with 2 × 2 contingency table and considered *p* < 0.05 as significant;

† Fisher Exact test.

**Table 4:** The primers, enzymatic incubation and polymerase chain reaction (PCR) cycling specification of the Leptin receptor gene Gln223Arg loci.

SNP ID	Amino acid changing region	Enzymatic digestion time temperature and volume	Cutting position	Forward and reverse primer	PCR product size (bp)	PCR cycling condition (time and temperature)	Reference
rs1137101	Gln223Arg	*Msp* I 45 min 37 °C 4 Unit	5′...C^C G G...3′ 3′...G G C^C...5′	5′-CAAACTCAACGACACTCTCCTT-3′ 5′-CTGAACTGACATTAGAGGTGAC-3′	80	5 min 95 °C 45 s 95 °C 30 s 57 °C 45 s 72 °C 30 cycle 10 min 72 °C 4 °C ∞	[37]

## References

[1] Nor M., Safiza N., Khor G.L., Shahar S., Kee C.C., Haniff J., Appannah G., Rasat R., Wong N.F., Zainuddin A.A. (2008). The Third National Health and Morbidity Survey (NHMS III) 2006, nutritional status of adults aged 18 years and above. Malays. J. Nutr.

[2] Almdal T., Scharling H., Jensen J.S., Vestergaard H. (2008). Higher prevalence of risk factors for type 2 diabetes mellitus and subsequent higher incidence in men. Eur. J. Intern. Med.

[3] Kumari M., Head J., Marmot M. (2004). Prospective study of social and other risk factors for incidence of type 2 diabetes in the Whitehall II study. Arch. Intern. Med.

[4] Meisinger C., Thorand B., Schneider A., Stieber J., Doring A., Lowel H. (2002). Sex differences in risk factors for incident type 2 diabetes mellitus: The MONICA Augsburg cohort study. Arch. Intern. Med.

[5] Njølstad I., Amesen E., Lund-Larsen P.G. (1998). Sex differences in risk factors for clinical diabetes mellitus in a general population: A 12-year follow-up of the Finnmark Study. Am. J. Epidemiol.

[6] Hebebrand J., Hinney A. (2009). Environmental and genetic risk factors in obesity. Child Adolesc. Psychiatr. Clin. N. Am.

[7] Morrish N.J., Wang S.L., Stevens L.K., Fuller J.H., Keen H. (2001). Mortality and causes of death in the WHO multinational study of vascular disease in diabetes. Diabetologia.

[8] Haffner S.M., Miettinen H., Stern M.P. (1997). Relatively more atherogenic coronary heart disease risk factors in prediabetic women than in prediabetic men. Diabetologia.

[9] Booth G.L., Kapral M.K., Fung K., Tu J.V. (2006). Recent trends in cardiovascular complications among men and women with and without diabetes. Diabetes Care.

[10] Alexander C.M., Landsman P.B., Teutsch S.M., Haffner S.M. (2003). NCEP-defined metabolic syndrome, diabetes, and prevalence of coronary heart disease among NHANES III participants age 50 years and older. Diabetes.

[11] Clément K., Vaisse C., Lahlou N., Cabrol S., Pelloux V., Cassuto D., Gourmelen M., Dina C., Chambaz J., Lacorte J.M. (1998). A mutation in the human leptin receptor gene causes obesity and pituitary dysfunction. Nature.

[12] Davies L., Marks J.L. (1994). Role of hypothalamic neuropeptide Y gene expression in body weight regulation. Am. J. Physiol.

[13] Mantzoros C.S., Moschos S.J. (1998). Leptin: In search of role(s) in human physiology and pathophysiology. Clin. Endocrinol.

[14] Garcia-Mayor R.V., Andrade M.A., Rios M., Lage M., Dieguez C., Casanueva F.F. (1997). Serum leptin levels in normal children: Relationship to age, gender, body mass index, pituitary-gonadal hormones, and pubertal stage. J. Clin. Endocrinol. Metab.

[15] Oksanen L., Mustajoki P., Kaprio J., Kainulainen K., Jaenne L., Peltonen L., Kontula K. (1996). Polymorphism of the β_3_-adrenergic receptor gene in morbid obesity. Int. J. Obes.

[16] Myers M.G., Leibel R.L., Seeley R.J., Schwartz M.W. (2010). Obesity and leptin resistance: Distinguishing cause from effect. Trends Endocrinol. Metab..

[17] Lahlou N., Clement K., Carel J.C., Vaisse C., Lotton C., Le Bihan Y., Lotton C., Basdevant A., Lebouc Y., Froguel P. (2000). Soluble leptin receptor in serum of subjects with complete resistance to leptin: Relation to fat mass. Diabetes.

[18] Burguera B., Couce M.E., Curran G.L., Jensen M.D., Lloyd R.V., Cleary M.P., Poduslo J.F. (2000). Obesity is associated with a decreased leptin transport across the blood-brain barrier in rats. Diabetes.

[19] Bougneres P. (2002). Genetics of obesity and type 2 diabetes: Tracking pathogenic traits during the predisease period. Diabetes.

[20] O’Rourke L., Yeaman S.J., Shepherd P.R. (2001). Insulin and leptin acutely regulate cholesterol ester metabolism in macrophages by novel signaling pathways. Diabetes.

[21] Mayes P.A., Murray R.K., Granner D.K., Mayers P.A., Rodwell V.W. (1988). Lipids of Physiologic Significance. Harper’s Biochemistry.

[22] Mendez-Sanchez N., Gonzalez V., King-Martinez A.C., Sanchez H., Uribe M. (2002). Plasma leptin and the cholesterol saturation of bile are correlated in obese women after weight loss. J. Nutr.

[23] Szapary P.O., Hark L.A., Burke F.M. (2002). The metabolic syndrome: A new focus for lifestyle modification. (CHD prevention). Patient Care.

[24] Shimabukuro M., Koyama K., Chen G., Wang M.Y., Trieu F., Lee Y., Newgard C.B., Unger R.H. (1997). Direct antidiabetic effect of leptin through triglyceride depletion of tissues. Proc. Natl. Acad. Sci. USA.

[25] Fairbrother U.L., Tankó L.B., Walley A.J., Christiansen C., Froguel P., Blakemore A.I. (2007). Leptin receptor genotype at Gln223Arg is associated with body composition, BMD, and vertebral fracture in postmenopausal Danish women. J. Bone Miner. Res.

[26] Wauters M., Mertens I., Rankinen T., Chagnon M., Bouchard C., van Gaal L. (2001). Leptin receptor gene polymorphisms are associated with insulin in obese women with impaired glucose tolerance. J. Clin. Endocrinol. Metab.

[27] Salopuro T., Pulkkinen L., Lindström J., Eriksson J.G., Valle T.T., Hämäläinen H., Ilanne-Parikka P., Keinänen-Kiukaanniemi S., Tuomilehto J., Laakso M. (2005). Genetic variation in leptin receptor gene is associated with type 2 diabetes and body weight: The Finnish Diabetes Prevention Study. Int. J. Obes.

[28] Liew S.F., Chuah H.S., Lau C.L., Lee C.H., Say Y.H. (2009). Prevalence of the leptin and leptin receptor gene variants and obesity risk factors among Malaysian University students of Setapak, Kuala Lumpur. Asian J. Epidemiol.

[29] Heo M., Leibel R.L., Fontaine K.R., Boyer B.B., Chung W.K., Koulu M., Karvonen M.K., Pesonen U., Rissanen A., Laakso M. (2002). A meta-analytic investigation of linkage and association of common leptin receptor (LEPR) polymorphisms with body mass index and waist circumference. Int. J. Obes. Relat. Metab. Disord.

[30] Constantin A., Costache G., Sima A.V., Glavce C.S., Vladica M., Popov D.L. (2010). Leptin G-2548A and leptin receptor Q223R gene polymorphisms are not associated with obesity in Romanian subjects. Biochem. Biophys. Res. Commun.

[31] Paracchini V., Pedotti P., Taioli E. (2005). Genetics of leptin and obesity: A HuGE review. Am. J. Epidemiol.

[32] Bender N., Allemann N., Marek D., Vollenweider P., Waeber G., Mooser V., Egger M., Bochud M. (2011). Association between variants of the leptin receptor gene (LEPR) and overweight: A systematic review and an analysis of the CoLaus study. PLoS One.

[33] Pluzhnikov A., Di Rienzo A., Hudson R.R. (2002). Inferences about human demography based on multilocus analyses of noncoding sequences. Genetics.

[34] Coelho M., Luiselli D., Bertorelle G., Lopes A.I., Seixas S., Destro-Bisol G., Rocha J. (2005). Microsatellite variation and evolution of human lactase persistence. Hum. Genet.

[35] Voight B.F., Kudaravalli S., Wen X., Pritchard J.K. (2006). A map of recent positive selection in the human genome. PLoS Biol.

[36] Grundy S.M. (2008). Metabolic syndrome pandemic. Arterioscler. Thromb. Vasc. Biol.

[37] Guizar-Mendoza J.M., Amador-Licona N., Flores-Martinez S.E., Lopez-Cardona M.G., Ahuatzin-Tremary R., Sanchez-Corona J. (2005). Association analysis of the Gln223Arg polymorphism in the human leptin receptor gene, and traits related to obesity in Mexican adolescents. J. Hum. Hypertens.

[38] Zeggini E., Scott L.J., Saxena R., Voight B.F., Marchini J.L., Hu T., de Bakker P.I., Abecasis G.R., Almgren P., Andersen G. (2008). Meta-analysis of genome-wide association data and large-scale replication identifies additional susceptibility loci for type 2 diabetes. Nat. Genet.

